# Postherpetic Neuralgia: Mechanisms, Risk Factors, and Stratified Management—A Narrative Review

**DOI:** 10.1002/cns.71002

**Published:** 2026-06-30

**Authors:** Fei Tang, Fukun Zhao

**Affiliations:** ^1^ Department of Pain Medicine Suiyang County Hospital of Traditional Chinese Medicine Guizhou People's Republic of China; ^2^ Department of Clinical Pharmacy Zunyi First People's Hospital (The Third Affiliated Hospital of Zunyi Medical University) Guizhou People's Republic of China

**Keywords:** herpes zoster, mechanisms, postherpetic neuralgia, risk factors, stratified management

## Abstract

**Background:**

In 2020, an estimated 14.9 million herpes zoster (HZ) cases occurred worldwide among adults aged ≥ 50 years; this number is projected to rise to 19.1 million by 2030. Postherpetic neuralgia (PHN), the most common complication of HZ, develops in 5% to > 30% of patients with HZ. Poorly managed PHN may cause persistent pain, functional impairment, psychological distress, and, in severe cases, suicide. This review summarizes the current evidence on PHN and proposes a tiered management framework.

**Methods:**

PubMed and Web of Science were searched for PHN‐related articles published from January 2011 to December 2025, with reference‐list screening. Studies were selected by clinical relevance and design. Management strategies were tiered by guideline support, regulatory approval, and PHN‐specific efficacy and safety evidence.

**Results:**

Pathogenesis involves viral reactivation, peripheral and central sensitization, and genetic modulation. Major risk factors include older age, severe acute pain, extensive rash, craniofacial/thoracic involvement, immunocompromise, comorbidities, and treatment delay. Management of PHN is tiered: Tier 1 treatments include gabapentinoids, the 5% lidocaine patch, tricyclic antidepressants, duloxetine, and the 8% capsaicin patch; Tier 2 treatments include botulinum toxin type A injections, pulsed radiofrequency, and temporary spinal cord stimulation (tSCS); and Tier 3 options include opioids and neuromodulation.

**Conclusions:**

PHN remains the most common and therapeutically challenging complication of HZ. Although a stratified framework for PHN management has been proposed, it still requires validation in clinical practice and further refinement to support more effective individualized treatment.

## Graphical abstract

The incidence of PHN is increasing, posing a substantial public health burden. Its pathogenesis involves VZV reactivation, peripheral and central sensitization, and genetic susceptibility, with key risk factors including older age, severe acute pain, extensive rash, immunocompromise, comorbidities, and delayed treatment. Management is tiered, progressing from first‐line pharmacologic therapy to interventional approaches, with opioids or neuromodulation reserved for refractory cases.

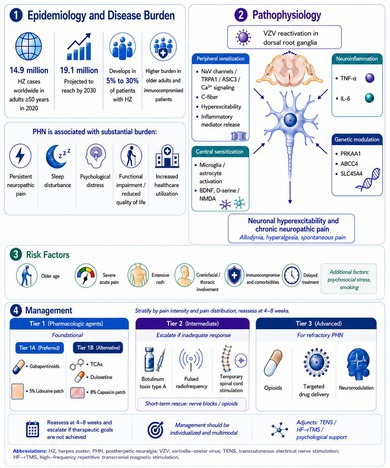

## 1 Introduction

Postherpetic neuralgia (PHN), the most common complication of herpes zoster (HZ), is a dermatomal pain syndrome that persists for at least 90 days after rash onset [1]. The lifetime risk of HZ is 36.5% (95% confidence interval [CI], 35.5–37.4) [2]. Among individuals with HZ, 5% to more than 30% develop PHN, with incidence increasing with advancing age [3, 4]. PHN‐related pain is often challenging to manage and may persist for months to years, occasionally lasting more than a decade [5]. Chronic pain, if inadequately managed, can impair physical functioning, psychological well‐being, social participation, and overall health, potentially leading to depression, anxiety, and, in severe cases, suicide [6, 7]. Among individuals with high‐impact chronic pain, such as PHN, 69.0% report anxiety and 65.8% report depression [8]. Patients with chronic pain and comorbid anxiety and/or depression experience substantial functional limitations: 69.4% report health‐related work impairment, 43.7% report difficulty performing household tasks independently, and 55.7% report reduced social engagement [9]. The economic burden is considerable; in China, the annual per‐patient direct medical cost for PHN is RMB 10,002 (US$1507), and the annual per‐patient indirect cost is RMB 28,025 (US$4221) [10]. Despite multiple regional recommendations, an evidence‐based, PHN‐specific guideline is still lacking. This narrative review summarizes current evidence regarding the epidemiology, pathophysiology, risk factors, and clinical management of PHN. It further proposes a tiered management framework for PHN based on neuropathic pain (NP) guidelines and PHN‐related clinical management evidence.

## 2 Methods

### 2.1 Literature Search Strategy and Study Selection

For this narrative review, relevant literature was defined as publications addressing the epidemiology, mechanisms, risk factors, prevention, and management of PHN. Literature retrieval was conducted in PubMed and Web of Science using MeSH terms, title/abstract terms, and topic terms related to PHN, including “postherpetic neuralgia,” “postherpetic pain,” “PHN,” “herpes zoster,” “shingles,” and “zoster.” The Web of Science search was limited to SCI‐EXPANDED and SSCI. English‐language articles published between January 1, 2011, and December 31, 2025, were included. Reference lists of relevant guidelines, systematic reviews, meta‐analyses, and original studies were also screened for additional publications. Studies were selected based on clinical relevance and design, prioritizing clinical practice guidelines, systematic reviews/meta‐analyses, randomized trials, and large observational studies. Landmark studies were included for historical or mechanistic context. A total of 81 publications were included: 8 randomized trials, 18 systematic reviews and meta‐analyses, 9 guidelines, 26 observational studies, 8 preclinical studies, and 12 narrative reviews (Table S1). As a narrative review, no formal risk‐of‐bias assessment or quantitative meta‐analysis was performed.

### 2.2 Operational Criteria for Tiered Classification of PHN Management Strategies

PHN management strategies were categorized according to prespecified operational criteria that integrated recommendations from 5 NP guidelines (Table S2), regulatory approval status, and PHN‐specific evidence on efficacy and safety. Tier 1 included interventions with broad guideline support, defined as those recommended as first‐ or second‐line treatments in at least three included guidelines. Tier 1A comprised interventions recommended as first‐line treatments in at least three guidelines and approved specifically for PHN. Tier 1B included two groups of interventions: those recommended as first‐line treatments in at least three guidelines but lacking PHN‐specific regulatory approval, and those approved for PHN but not meeting the criterion of concordant first‐line recommendation across at least three guidelines. Tier 2 included interventions supported by at least one guideline together with PHN‐specific evidence from a systematic review or meta‐analysis, or from at least two randomized controlled trials (RCTs), with additional consideration of the consistency of efficacy findings and safety profile. Tier 3 included investigational or lower‐evidence interventions supported mainly by limited uncontrolled studies, case series, or case reports.

## 3 Results

### 3.1 Epidemiology and Disease Burden

Seropositivity for the varicella‐zoster virus exceeds 95% in young adults, reflecting a widespread global risk of HZ and its complication, PHN [1]. In 2020, an estimated 14.9 million HZ cases occurred among adults aged ≥ 50 years worldwide, with projections rising to 19.1 million by 2030, largely attributable to population aging [11]. The global standardized incidence rate of HZ is 476.2 cases per 100,000 person‐years (95% CI, 468.3–483.8), and the median age at onset is 56.3 years (interquartile range, 43.0–68.7) [12]. Although the reported incidence of PHN following HZ varies substantially across regions, PHN remains the most common complication [3, 4, 13, 14, 15].

The risk of PHN is influenced primarily by age, immune function, and overall health status. PHN incidence increases with age, from 1.6 (95% CI, 1.2–2.0) per 100,000 person‐years in children aged < 10 years to 228.5 (95% CI, 219.7–236.6) in adults aged ≥ 71 years [12]. Globally, the standardized incidence rate of PHN is 57.5 per 100,000 person‐years (95% CI, 56.0–59.0), and the median age at onset is 65.9 years (interquartile range, 53.5–74.8) [12]. In adults aged 50–90 years, the cumulative risk is 6.9% (95% CI, 6.7–7.1) [14]. The incidence is higher in female individuals (70.3; 95% CI, 68.4–72.4) than in male individuals (44.5; 95% CI, 43.2–46.0) [12]. By race, the incidence is highest among White individuals (60.1 per 100,000 person‐years; 95% CI, 58.5–61.8), followed by Black (55.6; 95% CI, 53.4–57.8), Hispanic (49.7; 95% CI, 46.3–54.0), and Asian (44.3; 95% CI, 40.5–48.0) individuals [12]. Among immunocompromised patients, incidence ranges from 116.51 to 152.25 per 100,000 person‐years [16], reaching 490 among autologous hematopoietic stem cell transplant recipients [17]. Risk is also socially patterned; the risk of PHN is significantly lower in the highest quintile than in the lowest quintile (adjusted risk ratio, 0.68 [95% CI, 0.64–0.73]) [18].

Beyond differences in incidence across populations, PHN imposes a substantial clinical and societal burden. Sensory profiles revealed preserved sensation with thermal hyperalgesia in 27% (suggesting peripheral sensitization), small or large fiber loss in 25% (indicating neurodegeneration), and small fiber loss and mechanical allodynia in 48% (consistent with both peripheral neuropathy and central sensitization) [19]. Prodromal symptoms were reported by 56.7% of patients, with pain being the most common (82.4%) [3]. Comorbidities were present in 76.7% of patients. The most common comorbidities were hypertension (35.7%), diabetes (18.1%), osteoporosis (10.9%), and hypercholesterolemia (10.0%) [20]. PHN imposes a substantial functional and socioeconomic burden: a 26.2% reduction in workdays (absenteeism) and 48.4% reduction in on‐the‐job productive work time (presenteeism) [4]. Among employed individuals, 55.6% reported overall work impairment, and 51.1% reported activity limitations outside of work [10]. Healthcare utilization also increased significantly in the first year after PHN onset, with a mean of 16.30 outpatient visits, 0.40 emergency department visits, and 0.24 hospitalizations per person [21]. In summary, PHN poses a substantial public health burden by impairing quality of life and work capacity and increasing healthcare resource utilization.

**FIGURE 1:**
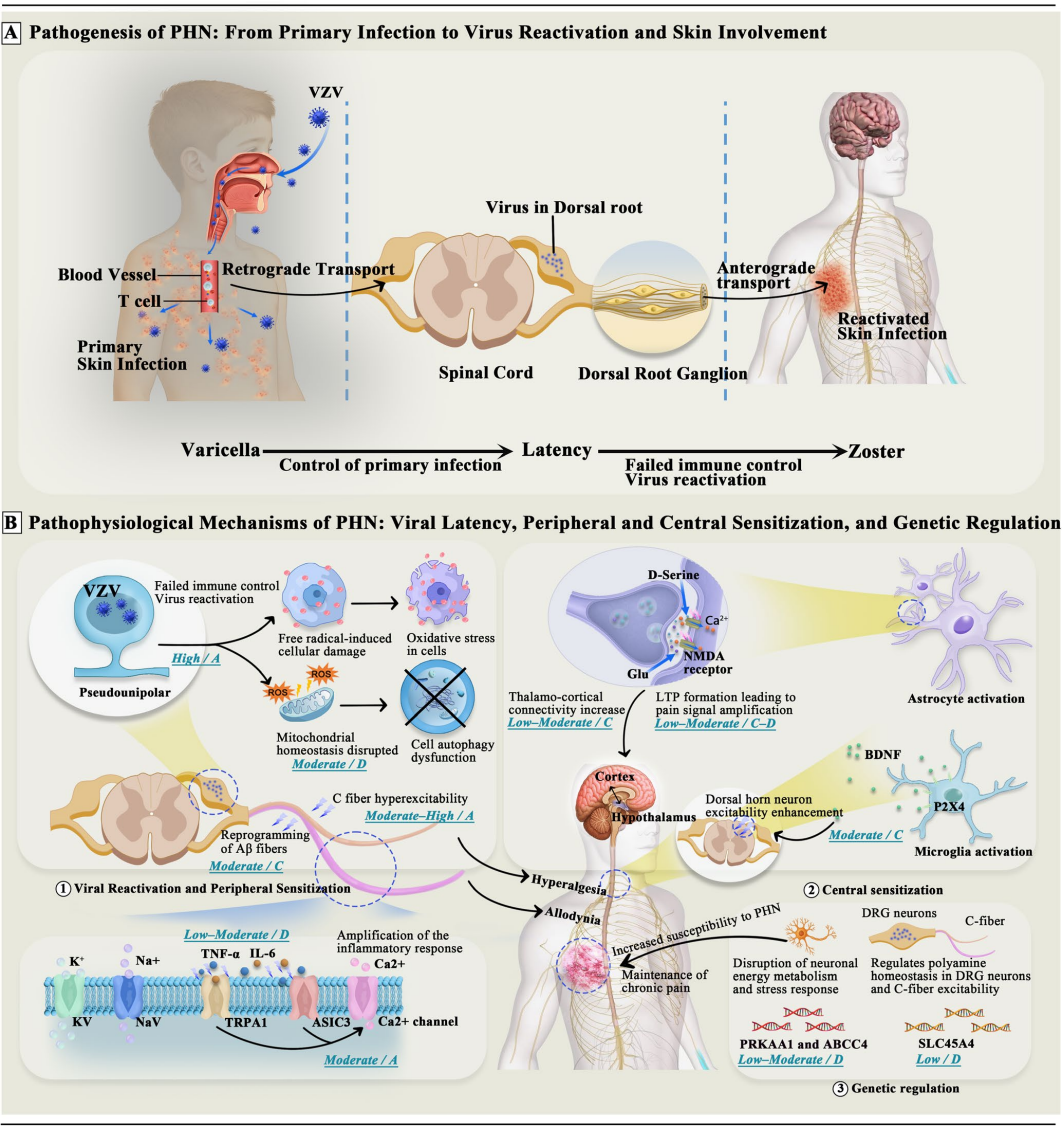
PHN: Integrated pathogenesis and pathophysiology mechanisms. (A) Varicella‐zoster virus (VZV) infection occurs through the respiratory mucosa, replicates locally, and spreads to the tonsils and regional lymph nodes. Infected T lymphocytes disseminate the virus via the bloodstream to the skin, producing a vesicular rash. During primary infection, VZV also travels retrogradely to the sensory ganglia, establishing latency. With impaired immunity, reactivation may occur, with anterograde spread along axons to the skin, causing vesicular eruptions in the corresponding dermatome. (B) This panel summarizes the major mechanisms involved in PHN, including VZV reactivation, mitochondrial dysfunction, oxidative stress, autophagy dysfunction, peripheral sensitization, central sensitization, and genetic regulation. Peripheral mechanisms include ion‐channel dysregulation, inflammatory mediator–driven amplification, C‐fiber hyperexcitability, and Aβ‐fiber reprogramming, contributing to hyperalgesia and allodynia. Central mechanisms include altered thalamo‐cortical connectivity, NMDA receptor–mediated pain signal amplification, astrocyte activation, and microglia‐related BDNF/P2X4 signaling, which enhance dorsal horn neuronal excitability and chronic pain maintenance. Genetic factors, including PRKAA1/ABCC4 and SLC45A4‐related pathways, may contribute to neuronal stress responses and PHN susceptibility. Blue italic labels indicate evidence strength/intervention‐potential grades. Grading criteria: Evidence strength was graded as High, Moderate, Low, or Very Low according to the strongest available evidence. Intervention potential was graded from A to D, ranging from PHN‐specific RCT/systematic review evidence to theoretical, preclinical, or risk‐stratification use only. ABCC4, ATP‐binding cassette subfamily C member 4; ASIC3, acid‐sensing ion channel 3; BDNF, brain‐derived neurotrophic factor; C‐fibers, C‐type sensory nerve fibers; DRG neurons, dorsal root ganglion neurons; LTP, long‐term potentiation; NMDA. *N*‐methyl‐d‐aspartate receptor; P2X4, purinergic receptor P2X4; PHN, postherpetic neuralgia; PRKAA1, protein kinase AMP‐activated catalytic subunit alpha 1; RCT, randomized clinical trial; SLC45A4, solute carrier family 45 member 4; TRPA1, transient receptor potential ankyrin 1; VZV, varicella‐zoster virus.

### 3.2 Pathophysiology

The pathogenesis of PHN is multifactorial, involving viral reactivation, peripheral and central sensitization, and genetic modulation (Figure 1). After immune surveillance failure, reactivation of latent varicella‐zoster virus in the dorsal root ganglia disrupts mitochondrial homeostasis, leading to autophagic dysfunction and oxidative stress that contribute to PHN onset [22, 23, 24]. Peripheral sensitization is characterized by abnormal sodium and potassium channel expression, which induces C‐fiber hyperexcitability and manifests as thermal and chemical hypersensitivity [25, 26]. Aβ‐fiber reprogramming further contributes to mechanical allodynia [27]. Inflammatory mediators, including tumor necrosis factor α and interleukin 6, activate nociceptive ion channels (e.g., TRPA1 and ASIC3) and alter Ca^2+^ signaling, amplifying inflammatory cascades and pain [28, 29, 30]. In central sensitization, microglia activation leads to brain‐derived neurotrophic factor release via P2X4 receptor signaling, enhancing dorsal horn neuron excitability [28, 31]. Astrocytic secretion of d‐serine co‐activates NMDA receptors, sustaining and amplifying nociceptive signaling, increasing thalamocortical connectivity, and perpetuating chronic pain [31, 32, 33]. In genetic modulation, variants in genes such as *PRKAA1* and *ABCC4* may impair neuronal energy metabolism and stress response pathways, potentiating PHN progression [34, 35, 36]. In contrast, SLC45A4, a polyamine transporter, regulates polyamine homeostasis in dorsal root ganglion neurons and C‐fiber excitability, and its variants may influence susceptibility to and severity of chronic pain [37].

### 3.3 Risk Factors

Age is a major determinant of PHN; the risk increases with age and is markedly higher in adults aged ≥ 50 years [14, 38]. Acute pain severity during the acute phase of HZ is another strong predictor, with higher intensity linked to increased risk of PHN [38]. The extent and severity of the rash are also correlated with PHN development, as patients with widespread or severe localized lesions are more likely to experience chronic NP [38]. Certain anatomical sites further influence PHN risk: HZ involving the craniofacial region or thoracic dermatomes is associated with a higher risk of PHN, whereas cervical or lumbar involvement confers a lower risk [38, 39]. Immunocompromised individuals, including those receiving immunosuppressive therapy or with underlying immunodeficiency disorders, face a markedly higher risk of PHN [38]. Comorbidities (e.g., diabetes mellitus, chronic obstructive pulmonary disease); psychosocial factors (e.g., adverse emotional states, personality disorders); and unhealthy lifestyle behaviors (e.g., smoking) are associated with an increased risk of PHN (Table 1) [38]. Delayed treatment substantially increases risk. Initiating therapy > 3 days after rash onset nearly doubles the risk (odds ratio [OR], 1.86; 95% CI, 1.13–3.07) [38], whereas for moderate‐to‐severe acute pain, delaying analgesic treatment for > 14 days confers a four‐fold higher risk (OR, 4.11; 95% CI, 1.69–9.92) [40]. Mendelian randomization analyses suggest that specific gut microbiota (e.g., *Eubacterium rectale* group) increase the risk of HZ (OR, 1.36; *p* = 0.025) and PHN (OR, 3.15; *p* = 0.035) [41]. Genetically predicted higher *N*‐acetyl‐aspartyl‐glutamate levels are associated with reduced PHN risk (OR, 0.83; 95% CI, 0.76–0.91; *p* = 2.68 × 10^−5^) [42]. Additional biological markers associated with PHN risk include immunological markers (e.g., elevated neutrophil‐to‐lymphocyte ratio and reduced number of CD8^+^ T cells), nerve injury‐related proteins (e.g., neuron‐specific enolase and myelin basic protein), genetic markers (e.g., *ABCC4* rs4773840 SNP), altered metabolomic/proteomic signatures (e.g., tryptophan dysregulation), and neuroimaging findings (e.g., increased mean white matter diffusivity) [43].

**TABLE 1:** Risk factors of Postherpetic Neuralgia from a meta‐analysis [38].

Risk factor category	Risk factor	OR	95% CI	*I* ^2^	*p*	Number of studies
Demographic characteristics						
	Age (per 10‐year increase)	1.52	1.40–1.64	84.2%	< 0.001	17
	Sex (female vs. male)	1.13	0.99–1.29	94.0%	0.072	20
Skin lesion‐related risks						
	Periocular region of the eye	1.96	1.75–2.20	0.0%	< 0.001	4
	Thoracic nerve region	3.59	1.67–7.72	0.0%	< 0.001	2
	Nontrunk region	2.20	1.55–3.13		< 0.001	1
	Upper arm	3.46	1.10–10.89		0.034	1
	Left side of the body	2.62	1.19–5.78		0.017	1
	Cervical	0.80	0.21–2.99		0.740	1
	Lumbar	1.29	0.61–2.74	63.1%	0.507	3
	Lesion area > 5% of the body surface area	5.66	1.63–19.66	92.7%	0.006	2
	Severe skin lesions	2.67	1.96–3.64	42.3%	< 0.001	10
	Prodromal pain	1.92	1.43–2.58	36.9%	< 0.001	15
	Severity of acute‐phase pain (binary classification)	2.53	2.10–3.06	0.0%	< 0.001	13
	Severity of acute‐phase pain (continuous data)	1.09	1.03–1.16	82.4%	0.003	10
	Severity of acute‐phase pain (graded data)	3.25	2.12–4.96	56.3%	< 0.001	4
Comorbidity‐associated risk						
	Severe immunosuppression	2.08	1.69–2.57	0.0%	< 0.001	3
	Autoimmune diseases	1.60	1.11–2.31	82.8%	0.011	2
	Diabetes	1.78	1.54–2.07	86.7%	< 0.001	20
	Adverse emotional states	1.40	1.07–1.84	48.1%	0.014	3
	Personality disorders	1.10	1.02–1.18	0.0%	0.019	2
	Specific comorbidities (e.g., COPD, asthma)	1.35	1.12–1.63	95.6%	0.001	6
	Smoking	1.77	1.04–2.99	70.3%	0.034	3
Treatment‐related risk						
	Immunosuppressive therapy	1.96	0.22–17.12	79.2%	0.544	2
	Glucocorticoid use	0.61	0.22–1.70	71.4%	0.347	3
	Initial treatment time (≥ 3 days vs. < 3 days)	1.86	1.13–3.07	76.4%	0.015	5

*Note:* OR: OR > 1 indicates increased risk in the exposed group, OR < 1 indicates decreased risk, and OR ≈1 suggests similar risk between groups. 95% CI: A 95% CI that includes 1 suggests the risk factor may not have a statistically significant effect. *I* ^2^: Measures variability across studies. *I* ^2^ > 50% indicates substantial heterogeneity. *p*‐value: *p* < 0.05 indicates statistical significance; *p* ≥ 0.05 suggests no significance. Number of studies: A larger number of studies strengthens the credibility of the conclusion.

Abbreviations: CI, confidence interval; COPD, chronic obstructive pulmonary disease; *I* ^2^, heterogeneity index; OR, odds ratio.

### 3.4 Management

**FIGURE 2:**
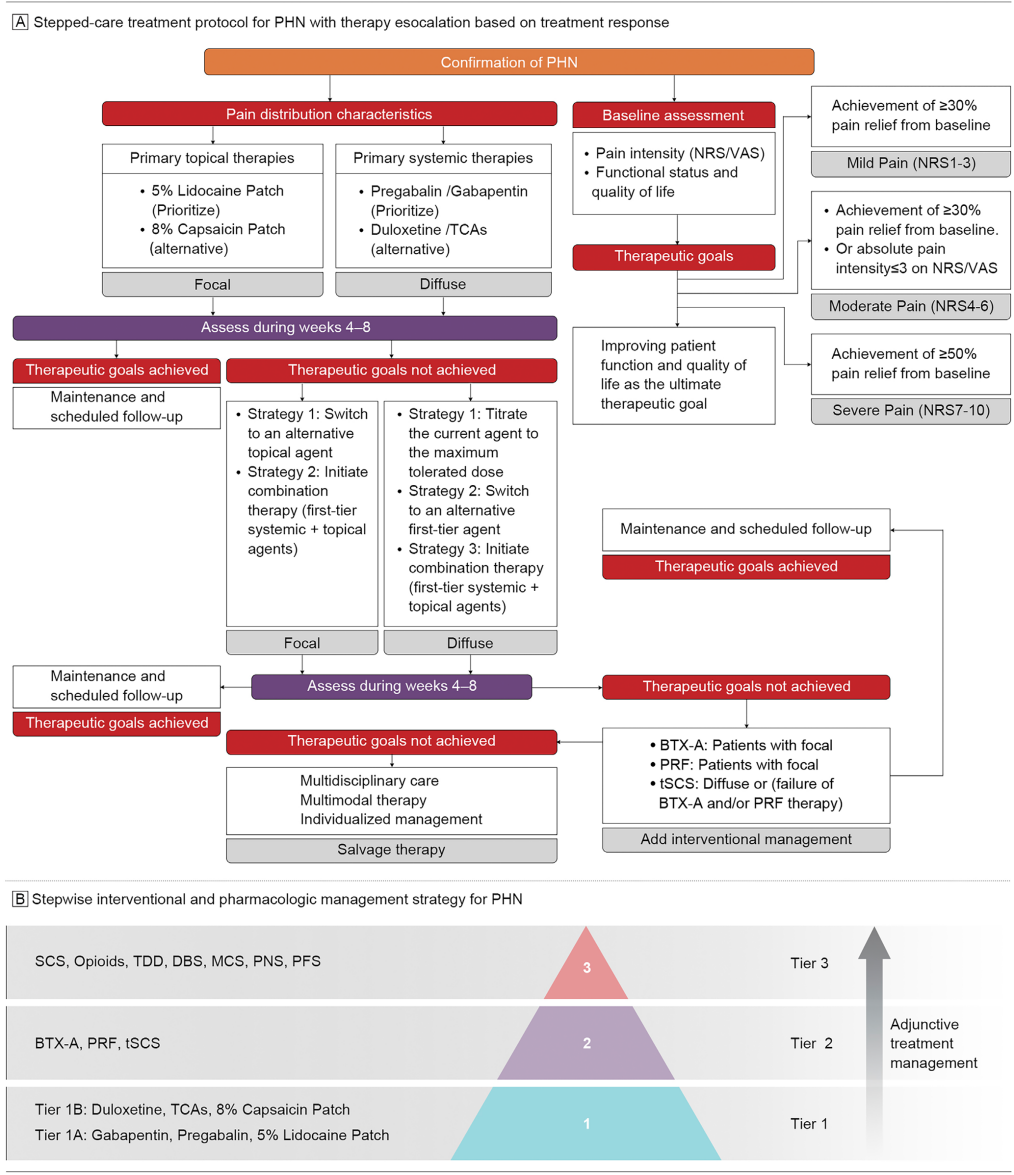
Management and treatment strategies for PHN. (A) Initial therapy is selected according to pain distribution (focal or diffuse) and baseline assessment. Focal pain is treated with topical agents, including a 5% lidocaine patch (preferred) or an 8% capsaicin patch (alternative), whereas diffuse pain is treated with systemic agents, including pregabalin or gabapentin (preferred), with duloxetine or tricyclic antidepressants (TCAs) as alternatives. Baseline assessment includes pain intensity (NRS/VAS), functional status, and quality of life. Therapeutic targets are defined by baseline pain severity: ≥ 30% pain relief for mild pain, ≥ 30% pain relief or pain score ≤ 3 for moderate pain, and ≥ 50% pain relief for severe pain. Treatment response is reassessed after 4–8 weeks. If goals are unmet, escalation includes switching, dose titration, combination therapy, and, if needed, interventional treatment with BTX‐A, PRF, or tSCS. Refractory cases may require multidisciplinary and individualized salvage management. (B) Tier 1 includes Tier 1A agents (gabapentin, pregabalin, and 5% lidocaine patch) and Tier 1B agents (duloxetine, TCAs, and 8% capsaicin patch). Tier 2 includes BTX‐A, PRF, and tSCS. Tier 3 includes spinal cord stimulation (SCS), opioids, transdermal drug delivery (TDD), deep brain stimulation (DBS), motor cortex stimulation (MCS), peripheral nerve stimulation (PNS), and pain‐free status (PFS)–oriented advanced management strategies. Overall, PHN management follows a stepwise escalation model from first‐line pharmacologic therapy to adjunctive interventional and advanced treatments. BTX‐A, botulinum toxin A; DBS, deep brain stimulation; MCS, motor cortex stimulation; NRS/VAS, numerical rating scale/visual analog scale; PFS, pain‐free status; PHN, postherpetic neuralgia; PNS, peripheral nerve stimulation; PRF, pulsed radiofrequency; SCS, spinal cord stimulation; TCA, tricyclic antidepressants; TDD, transdermal drug delivery; tSCS, temporary spinal cord stimulation.

Management of PHN, a refractory subtype of NP, remains challenging. Fewer than 50% of patients achieve ≥ 50% pain relief [1]. No PHN‐specific guidelines exist; clinicians rely on broader NP recommendations (Table 2) [44, 45, 46, 47, 48]. Key principles are: (1) stratifying therapy by baseline pain intensity (mild, moderate, severe) and pain distribution (focal or diffuse) [46, 47, 48]; (2) setting realistic treatment goals, such as ≥ 30% or ≥ 50% pain reduction or a Numeric Rating Scale (NRS)/Visual Analog Scale (VAS) score of ≤ 3 [47, 48]; (3) prioritizing evidence‐based, low‐risk options to optimize safety and tolerability [44]; and (4) assessing at 4–8 weeks (earlier if clinically indicated); if pain is intolerable or the regimen is poorly tolerated, promptly modifying or escalating therapy [44]. Figure 2 shows the overall approach (A) and stepwise stratification (B). Table 3 summarizes the pharmacologic and interventional evidence [49, 50, 51, 52, 53, 54, 55, 56, 57, 58].

**TABLE 2:** Stepwise therapies for neuropathic pain: Alignment and variations in international clinical guidelines.

Guideline source	Treatment recommendations
Comprehensive Algorithm on Management of NP [44].	First‐line: Nonpharmacological: multidisciplinary care (e.g., psychology, physiotherapy, exercise therapy, and massage therapy). Pharmacological: TCAs (amitriptyline, nortriptyline), SNRIs (duloxetine, venlafaxine), gabapentinoids (gabapentin, pregabalin), and topical agents (5% lidocaine patch, 8% capsaicin patch). Second‐line: Combination therapy (two first‐line agents), tramadol, and tapentadol. Third‐line: Pharmacological (requires specialist supervision): SSRIs, anticonvulsants (lamotrigine, carbamazepine, topiramate, sodium valproate), and NMDA receptor antagonists. Interventional: epidural injections (nonparticulate steroids recommended), PRF, and sympathetic nerve blocks (limited to CRPS). Fourth‐line: Neurostimulation (e.g., SCS and DRG‐S). Fifth‐line: Strong opioids (e.g., oxycodone, morphine, methadone, levorphanol). Sixth‐line: Targeted drug delivery (e.g., intrathecal drug delivery systems).
National Institute for Health and Care Excellence (NICE) [45].	First‐line: Amitriptyline, duloxetine, gabapentin, or pregabalin. Second‐line: Rescue therapy: tramadol is restricted to acute pain management. Topical therapy: capsaicin cream for patients who are intolerant of, or have contraindications to, oral agents.
German Neurological Society (DGN) [46].	First‐line: Gabapentinoids (gabapentin, pregabalin), TCAs (e.g., amitriptyline, imipramine, clomipramine), and duloxetine. Second‐line: 5% lidocaine patch (first‐line for PHN) and 8% capsaicin patch. Third‐line: Tramadol, strong opioids, and BTX‐A (for focal pain in specialized centers). Note: Psychotherapeutic approaches may be used for neuropathic pain of any etiology. Multimodal pain therapy is an important option for difficult‐to‐treat chronic neuropathic pain.
International Association for the Study of Pain French Chapter (SFETD) and French Society of Neurolog (SFN) [47].	First‐line: Pharmacological: duloxetine, venlafaxine, gabapentin, TCAs (e.g., nortriptyline, imipramine), and lidocaine patch (for peripheral NP). Nonpharmacological: TENS. Second‐line: Pharmacological: pregabalin, 8% capsaicin patch (for peripheral NP), tramadol, and combination therapy (TCA or SNRI + gabapentinoid). Interventional: BTX‐A (subcutaneous injection). Third‐line: Pharmacological: strong opioids (e.g., morphine, oxycodone) and combination therapy (TCA, SNRI, or gabapentinoid + opioid). Nonpharmacological: HF‐rTMS over M1 and SCS (for FBSS or DPN). Note: PRF is weakly recommended for thoracic PHN (moderate‐quality evidence). CBT and mindfulness are weakly recommended as add‐on therapies for NP (moderate‐quality evidence).
International Association for the Study of Pain (NeuPSIG) [48].	First‐line: TCAs (e.g., amitriptyline), gabapentinoids (pregabalin, gabapentin), and SNRIs (e.g., duloxetine, venlafaxine). Second‐line: Capsaicin patch, capsaicin cream, and lidocaine patch. Third‐line: BTX‐A (subcutaneous injection), rTMS over M1, and opioids.

Abbreviations: BTX‐A, botulinum toxin A; CBT, cognitive behavioral therapy. CRPS, complex regional pain syndrome; DPN, diabetic peripheral neuropathy; DRG‐S, dorsal root ganglion stimulation; FBSS, failed back surgery syndrome; HF, high‐frequency; M1, primary motor cortex; NMDA, *N*‐Methyl‐d‐Aspartate; NP, neuropathic pain; PRF, pulsed radiofrequency; rTMS, repetitive transcranial magnetic stimulation; SCS, spinal cord stimulation; SNRIs, serotonin‐norepinephrine reuptake inhibitors; TCAs, tricyclic antidepressants; TENS, transcutaneous electrical nerve stimulation.

**TABLE 3:** Efficacy of pharmacological and interventional therapies for postherpetic neuralgia: Data from meta‐analyses and randomized controlled trials.

Type of intervention	Follow‐up duration	Outcomes	Supporting studies
Oral drug management			
Gabapentin ≥ 1200 mg vs. placebo	4–12 w	≥ 50% PIR, 1.7 (1.4–2.0) favoring Gabapentin; 7 trials ≥ 30% PIR, 1.4 (1.1–1.7) favoring Gabapentin; 2 trials PGIC very much improved, 2.7 (1.5–4.8) favoring Gabapentin; 2 trials PGIC much or very much improved, 1.3 (1.2–1.5) favoring Gabapentin; 7 trials	Wiffen et al. [49].
Pregabalin 300 mg daily vs. placebo	≥ 8 w	≥ 50% PIR, 2.52 (1.86–3.42) favoring Pregabalin; 4 trials ≥ 30% PIR, 2.05 (1.63–2.57) favoring Pregabalin; 3 trials PGIC much or very much improved, 2.13 (1.54–2.94) favoring Pregabalin; 3 trials	Derry et al. [50].
Pregabalin 600 mg daily vs. placebo	≥ 8 w	≥ 50% PIR, 2.66 (2.04–3.48) favoring Pregabalin; 4 trials ≥ 30% PIR, 2.53 (2.01–3.18) favoring Pregabalin; 3 trials PGIC much or very much improved, 2.27 (1.33–3.89) favoring Pregabalin; 1 trial	Derry et al. [50].
Mirogabalin 15 mg daily vs. placebo	14 w	ADPS, −0.41 (−0.74 to −0.07) favoring Mirogabalin ADPS responder rate (≥ 50%), 1.20 (0.75–1.93) favoring Mirogabalin ADPS responder rate (≥ 30%), 1.54 (1.03–2.29) favoring Mirogabalin	Kato et al. [51].
Mirogabalin 20 mg daily vs. placebo	14 w	ADPS, −0.47 (−0.81 to −0.14) favoring Mirogabalin ADPS responder rate (≥ 50%), 1.48 (0.93–2.34) favoring Mirogabalin ADPS responder rate (≥ 30%), 1.52 (1.02–2.27) favoring Mirogabalin	Kato et al. [51].
Mirogabalin 30 mg daily vs. placebo	14 w	ADPS, −0.77 (−1.10 to −0.44) favoring Mirogabalin ADPS responder rate (≥ 50%), 1.63 (1.04–2.56) favoring Mirogabalin ADPS responder rate (≥ 30%), 1.81 (1.21–2.69) favoring Mirogabalin	Kato et al. [51]
Crisugabalin 40 mg daily vs. placebo	12 w	ADPS, −1.1 (−1.6 to −0.7, *p* < 0.001) favoring Crisugabalin ADPS responder rate (≥ 50%), 3.03 (1.50–6.13) favoring Crisugabalin ADPS responder rate (≥ 30%), 3.58 (1.90–6.76) favoring Crisugabalin	Zhang et al. [52].
Crisugabalin 80 mg daily vs. placebo	12 w	ADPS, −1.5(−2.0 to −1.0, *p* < 0.001) favoring Crisugabalin ADPS responder rate (≥ 50%), 3.84 (1.88–7.87) favoring Crisugabalin ADPS responder rate (≥ 30%), 2.93 (1.58–5.44) favoring Crisugabalin	Zhang D et al. [52].
Topical drug management			
5% Lidocaine Patch vs. placebo			Wang et al. [53].
	1 w	Change in VAS score: 5.68 ± 7.73 vs. 3.09 ± 6.19, (*p* = 0.0045) favoring Lidocaine Patches	
	2 w	Change in VAS score: 9.72 ± 10.09 vs. 6.80 ± 10.34, (*p* = 0.0366) favoring Lidocaine Patches	
	4 w	Change in VAS score: 14.01 ± 14.35 vs. 9.36 ± 12.03, (*p* = 0.0088) favoring Lidocaine Patches	
8% topical capsaicin vs. 0.04% topical capsaicin			Derry et al. [54].
	2–8 w	≥ 30% PIR, 1.3 (1.1–1.5) favoring 8% topical capsaicin; 4 trials	
	2–12 w	≥ 30% PIR, 1.3 (1.1–1.5) favoring 8% topical capsaicin; 3 trials	
	2–8 w	≥ 50% PIR, 1.4 (1.1–1.9) favoring 8% topicalcapsaicin;3 trials	
	2–12 w	≥ 50% PIR, 1.3 (1.0–1.7) favoring 8% topical capsaicin; 2 trials	
	8 w	PGIC much or very much improved, 1.4 (1.1–1.8) favoring 8% topical capsaicin; 2 trials	
	12 w	PGIC much or very much improved, 1.6 (1.2–2.0) favoring 8% topical capsaicin; 2 trials	
Interventional management			
Subcutaneous injection of BTX‐A vs. analgesics			Wang et al. [55]
	2 w	MD, −1.91 (2.63 to −1.20) favoring BTX‐A; 7 trials	
	4 w	MD, −1.69 (−2.69 to −0.68) favoring BTX‐A; 10 trials	
	8 w	MD, −1.66 (−2.20 to −1.12) favoring BTX‐A; 8 trials	
	12 w	MD, −1.83 (−2.70 to −0.96) favoring BTX‐A; 6 trials	
	24 w	MD, −1.07 (−1.16 to −0.99) favoring BTX‐A; 3 trials	
	8–12 w	≥ 50% PIR, 3.17 (2.14–4.72) favoring BTX‐A; 9 trials	
PRF vs. Sham Treatment or Oral Medicine Group			Wu et al. [56].
	2–3 days	WMD: −2.82 (−5.08 to −0.55) favoring PRF; 2 trials	
	1 w	WMD: −2.95 (−4.53 to −1.37) favoring PRF; 3 trials	
	2 w	WMD: −3.17 (−4.11 to −2.23) favoring PRF; 4 trials	
	4 w	WMD: −2.59 (−3.40 to −1.79) favoring PRF; 4 trials WMD: −3.02 (−4.17 to −1.88) favoring PRF; 4 trials	
	8 w		
	6 m	WMD: −1.94 (−2.85 to −1.03) favoring PRF; 2 trials	
tSCS vs. PRF			Abbas A et al. [57].
	1 w	MD: −0.88 (−2.01–0.25) no significant difference; 4 trials RD: 0.11(−0.05–0.27) no significant difference; 3 trials	
	1 m	MD: −0.98 (−1.77 to −0.19) favoring tSCS; 4 trials RD: 0.14(−0.10 to −0.38) no significant difference; 3 trials	
	3 m	MD: −1.34 (−2.59 to −0.09) favoring tSCS; 4 trials RD: 0.37 (0.10–0.63) favoring tSCS; 3 trials	
	6 m	MD: −1.27 (−2.30 to −0.23) favoring tSCS; 4 trials RD: 0.26 (0.13–0.39) favoring tSCS; 3 trials	
	12 m	MD: 0.39 (−0.64 to −1.42) no significant difference; 1 trial RD: 0.37(0.16–0.59) favoring tSCS; 1 trial	

Abbreviations: ADPS, average daily pain score; BTX‐A, botulinum toxin A; d, days; m, monthst; MD, mean difference; PGIC, patient global Impression of change; PIR, pain intensity reduction; PRF, pulsed radiofrequency; RD, risk difference; SCS, temporary spinal cord stimulation; VAS, visual analogue scale; w, weeks; WMD, weighted mean differences.

#### 3.4.1 Tier 1 Treatments

Tier 1 treatments are divided into Tier 1A (preferred) and Tier 1B (alternative options). Tier 1A includes gabapentinoids (e.g., gabapentin and pregabalin) and 5% lidocaine patch. Tier 1B includes tricyclic antidepressants (TCAs; e.g., amitriptyline, nortriptyline, and desipramine), duloxetine, and 8% capsaicin patch.

##### 3.4.1.1 Gabapentinoids

Gabapentin and pregabalin are approved for PHN by the United States Food and Drug Administration (FDA) [59] and are widely recommended as first‐line agents in NP guidelines [44, 46, 47, 48]. Gabapentinoids constitute a cornerstone of PHN management owing to their robust evidence base and widespread adoption in clinical practice.

A 2019 Cochrane review [50] reported dose‐dependent efficacy for pregabalin: at 300 mg/day, 32% of patients achieved ≥ 50% pain relief or Patient Global Impression of Change (PGIC) “very much improved” (number needed to treat [NNT] = 5.3; 95% CI, 3.9–8.1; 4 studies; 713 PHN patients) and at 600 mg/day, 41% achieved this outcome (NNT = 3.9; 95% CI, 3.1–5.5; 4 studies; 732 PHN patients). Adverse event risks increased with increasing dose (compared with placebo). At 300 mg/day: somnolence, 16.0% (placebo 5.5%; number needed to harm [NNH], 9.5); dizziness, 29.0% (placebo 8.1%; NNH = 4.8) [50]. At 600 mg/day: somnolence, 25.0% (5.8%; NNH = 5.2); dizziness, 35.0% (8.8%; NNH = 3.8) [50]. By comparison, gabapentin generally requires higher titrated doses; at ≥ 1200 mg/day, 32% of patients achieved ≥ 50% pain relief (NNT = 6.7; 95% CI, 5.4–8.7; 8 studies; 2260 PHN patients) [49]. Adverse events were also more frequent, compared with placebo: somnolence, 14.0% (placebo, 5.2%; NNH = 11); and dizziness, 19.0% (6.6%; NNH = 8) [49].

A 2022 meta‐analysis (14 RCTs; 3545 PHN patients) [60] found pregabalin superior to gabapentin for pain reduction (standardized mean difference [SMD], −1.65; 95% CI, −2.42 to −0.87), global impression improvement (Relative Risk [RR], 0.29; 95% CI, 0.20–0.39), and sleep interference (SMD, −0.83; 95% CI, −1.16 to −0.51; all *p* < 0.05). Adverse events, including dizziness, somnolence, peripheral edema, and weight gain, were more common with pregabalin (RR for gabapentin vs. pregabalin, 0.16; 95% CI, 0.09–0.23; *p* < 0.05) [60].

Newer gabapentinoids, such as mirogabalin (Japan, 2019) and cregabalin (China, 2024), demonstrated comparable efficacy to pregabalin in phase 3 trials [51, 52, 61]. A potential advantage may be improved tolerability, with lower rates of dizziness (15.5% with mirogabalin vs. 30.3% with pregabalin), peripheral edema (7.1% vs. 12.4%), and weight gain (5.2% vs. 16.9%) [51, 52]. However, long‐term efficacy, cost‐effectiveness, and head‐to‐head data remain limited.

##### 3.4.1.2 Antidepressants

Although not FDA‐approved for PHN, TCAs (e.g., amitriptyline, nortriptyline, and desipramine) and serotonin–norepinephrine reuptake inhibitors (SNRIs, e.g., duloxetine) are consistently recommended as first‐line treatments for NP in major guidelines [44, 46, 47, 48]. A 2023 Cochrane network meta‐analysis [62] of antidepressants (including amitriptyline, nortriptyline, duloxetine, and venlafaxine) found duloxetine to be the most effective across multiple outcomes (pain, function, sleep, and quality of life) for chronic pain, particularly NP; in pooled analyses of 16 RCTs (*n* = 4490), duloxetine 60 mg increased the odds of achieving ≥ 50% pain relief (OR, 1.91; 95% CI, 1.69–2.17; moderate certainty evidence).

Specifically, a 2025 double‐blind, randomized crossover trial [63] of patients with PHN (*n* = 220) showed no significant difference between duloxetine plus pregabalin and amitriptyline plus pregabalin: ≥ 50%, 25%–50%, and ≤ 25% pain relief occurred in 52%, 24%, and 7% vs. 48%, 21%, and 9% of patients, respectively (all *p* > 0.05). Sleep quality (assessed using the Pittsburgh Sleep Quality Index), depressive symptoms, and quality of life (assessed using Short Form‐36 Health Survey) were also comparable (all *p* > 0.05), whereas dry mouth was more frequent with amitriptyline than with duloxetine (26% vs. 11%; *p* = 0.008). Consistent with tolerability considerations, the 2025 NeuPSIG update published in *Lancet Neurology* reported treatment‐related withdrawal rates with active treatment compared with placebo of 9.7% vs. 4.8% for gabapentinoids, 11.9% vs. 3.9% for SNRIs, and 50.4% vs. 3.4% for TCAs in NP populations, with limited PHN‐specific data [48].

##### 3.4.1.3 Topical Agents

Topical therapies provide localized analgesia while minimizing systemic adverse effects and are especially appropriate for patients with localized pain or intolerance to systemic treatments [44, 48]. The 5% lidocaine patch and 8% capsaicin patch are FDA‐approved topical therapies for PHN [64, 65]. Major guidelines frequently recommend these as first‐ or second‐line options for NP [44, 46, 47, 48].

In a 2023 phase 3 RCT of 240 patients with PHN, the 5% lidocaine patch, compared with placebo, produced greater pain reduction at 4 weeks (mean change on the 0–100‐mm VAS, −14.01 [standard deviation, 14.35] vs. −9.36 [12.03]; *p* = 0.0088), a higher proportion achieving ≥ 30% pain relief (34.17% vs. 15.83%; *p* < 0.001), and similar adverse event rates (33.33% vs. 37.29%; *p* = 0.586) [53].

A 2017 Cochrane review [54] showed that, compared with placebo or active placebo, a single 60‐min application of the 8% capsaicin patch yielded higher global improvement at 12 weeks in patients with PHN (2 RCTs; *n* = 571; PGIC, 39% vs. 25%; NNT, 7.0; 95% CI, 4.6–15; moderate quality evidence); safety analyses (mixed NP including PHN, 7 RCTs; *n* = 1993; moderate quality evidence) showed no significant difference in serious systemic adverse events (RR, 1.14; 95% CI, 0.70–1.86), although transient local skin reactions were common.

A 2020 network meta‐analysis [66] (12 studies; *n* = 1563) compared 7 topical PHN interventions. The 5% lidocaine patch was significantly more effective than placebo (SMD, −1.31; 95% CI, −2.11 to −0.50; *p* = 0.01) and was ranked first for efficacy, followed by aspirin/diethyl ether mixture, high‐concentration capsaicin patch (8%), indomethacin, diclofenac, low‐concentration capsaicin, and placebo.

##### 3.4.1.4 Combination Pharmacotherapy

The 2020 French Society for the Study and Treatment of Pain (SFETD) and French NP Society (SFN) guidelines recommend an antidepressant (TCA or SNRI) plus a gabapentinoid as second‐line therapy for NP and an antidepressant or a gabapentinoid plus an opioid as third‐line therapy [47]. However, a 2023 meta‐analysis [67] found no significant benefit in achieving at least 30% pain relief with these combinations compared with the corresponding monotherapy for NP: opioid plus gabapentinoid compared with opioid alone (3 studies; *n* = 548; RR, 1.06; 95% CI, 0.80–1.40), opioid plus antidepressant compared with opioid alone (2 studies; *n* = 214; RR, 1.22; 95% CI, 0.97–1.52), and gabapentinoid plus antidepressant compared with gabapentinoid alone (3 studies; *n* = 527; RR, 1.40; 95% CI, 0.93–2.10). Similarly, safety outcomes showed no benefit; rather, combining opioids with gabapentinoids may increase adverse events [67]. In a case‐crossover study of 1,021,885 opioid users, analysis of 232 opioid‐related deaths (ORDs) found that the concomitant use of gabapentinoids, compared with no concomitant use, was associated with an approximately 132% increase in the odds of ORD (adjusted odds ratio [aOR] = 2.32; 95% CI, 1.01–5.33; *p* < 0.05) [68]. In contrast, combining a systemic first‐line agent with topical therapy appears advantageous. In a two‐phase trial involving 311 patients with NP [69], patients with insufficient response to pregabalin (NRS‐3 > 4) received add‐on lidocaine patch (PL; *n* = 44), and those with insufficient response to the lidocaine patch received add‐on pregabalin (LP; *n* = 57). After combination therapy, pain intensity decreased substantially in both groups (approximately 48%): the NRS reduced from 5.7 to 4.0 in the PL group and from 6.1 to 3.6 in the LP group, without a notable increase in adverse events [69]. In the pregabalin dose‐reduction sub‐study, more than 95% of patients reduced or discontinued pregabalin; among 21 patients with diabetic peripheral neuropathy, 20 reduced pregabalin by ≥ 150 mg/day, and all 10 patients with PHN discontinued pregabalin [69].

#### 3.4.2 Tier 2 Treatments

If Tier 1 treatment is inadequate, Tier 2 options may be considered, including botulinum toxin type A (BTX‐A) injection, pulsed radiofrequency (PRF), and temporary spinal cord stimulation (tSCS). Opioids and nerve blocks may also be used for short‐term pain relief.

##### 3.4.2.1 Botulinum Toxin Type A

For focal NP, BTX‐A or PRF may be prioritized. Several guidelines recommend subcutaneous BTX‐A as a second‐ or third‐line option for NP [46, 47, 48]. In a 2024 meta‐analysis of 14 RCTs including 1358 patients with PHN, BTX‐A reduced VAS pain scores at 2–24 weeks compared with conventional analgesics (mean differences [MDs], −1.07 to −1.91; all *p* < 0.00001) and increased the likelihood of achieving ≥ 50% pain reduction (OR, 3.17; 95% CI, 2.14–4.72) [55]. Adverse events were infrequent and mild, mainly transient injection‐site pain or dizziness (OR, 1.25; 95% CI, 0.43–3.61; *p* = 0.69) [55].

##### 3.4.2.2 Pulsed Radiofrequency

The 2019 review, *A Comprehensive Algorithm for Management of NP (CAMP)*, classified PRF as a third‐line therapy for NP, and the 2020 SFETD and SFN guidelines issued only weak recommendations for its use in thoracic PHN [44, 47]. A 2020 meta‐analysis [56] of patients with PHN (6 RCTs; *n* = 420) reported that PRF, compared with sham procedures or oral medications, significantly reduced pain, with effects evident within 2–3 days and persisting up to 6 months (weighted MD, −1.94 [95% CI, −2.85 to −1.03]; *p* < 0.0001; based on 2 trials; *n* = 135). No serious adverse events were reported; minor events (e.g., nausea and dizziness) were infrequent [56].

##### 3.4.2.3 Temporary Spinal Cord Stimulation

For diffuse pain or after failure of other Tier 2 interventions (BTX‐A and/or PRF), tSCS may be considered. The 2019 *CAMP* classified tSCS as fourth‐line therapy for NP, and the 2020 SFETD and SFN guidelines recommended it as third‐line therapy for failed back surgery syndrome or diabetic polyneuropathy, but not specifically for PHN [44, 47]. More recent evidence suggests greater benefit. A 2025 meta‐analysis [57] (4 RCTs; 261 PHN patients) demonstrated that tSCS was superior to PRF in achieving > 50% pain relief at 3, 6, and 12 months (risk difference [RD] = 0.37; 95% CI, 0.10–0.63; *p* = 0.007; RD = 0.26; 95% CI, 0.13–0.39; *p* < 0.001; and RD = 0.37; 95% CI, 0.16–0.59; *p* < 0.001). Another 2025 meta‐analysis [58] (7 RCTs; 412 PHN patients) reported fewer adverse events with tSCS than with PRF (RR, 0.17; 95% CI, 0.05–0.62; *p* = 0.007) but higher costs and greater procedural complexity.

##### 3.4.2.4 Opioids and Nerve Blocks

The 2019 *CAMP* classified weak opioids (e.g., tramadol and tapentadol) as a pharmacologic rescue therapy for acute exacerbations of NP [44]. Nerve blocks can provide rapid pain relief when opioid analgesia is inadequate and may be considered before initiating opioid therapy. In a 2018 RCT of 30 patients with PHN, nerve block produced a significantly greater reduction in 12‐h resting VAS pain scores compared with gabapentin plus tramadol (*p* < 0.001), with scores decreasing from 8.07 (0.96) to 3.40 (0.63) in the nerve block group and from 7.73 (0.88) to 4.80 (0.77) in the control group [70]. A 2024 evidence‐based update further supports nerve blocks as a short‐term analgesic strategy in PHN [71].

#### 3.4.3 Tier 3 Treatments

For refractory cases that do not respond to Tier 2 treatments, Tier 3 treatments may be warranted. Guiding principles [44, 47] include (1) multidisciplinary care involving pain medicine, neurology, psychiatry, and other relevant specialties; (2) multimodal therapy integrating pharmacological, interventional, and neuromodulation approaches; and (3) individualized management based on disease status, patient preferences, and available resources. Within this framework, Tier 3 options include opioids, targeted drug delivery (TDD), and neuromodulation (e.g., spinal cord, deep brain, motor cortex, peripheral nerve, and peripheral field stimulation).

##### 3.4.3.1 Spinal Cord Stimulation

The 2019 *CAMP* classifies spinal cord stimulation as fourth‐line therapy for NP [44]. Eligible patients have a chronic NP duration of ≥ 6 months and a VAS score ≥ 5/10 despite failure of appropriate standard therapies. A 5–7‐day percutaneous trial of stimulation should precede implantation; if ≥ 50% pain relief is achieved, permanent implantation may be considered [44, 72]. For PHN, evidence regarding its efficacy and safety is limited, consisting mainly of small nonrandomized studies and case series [73].

##### 3.4.3.2 Opioids

The 2019 *CAMP* recommends low‐dose opioids (morphine‐equivalent dose [MED] ≤ 50 mg/day) as fifth‐line therapy for NP and advises against MED > 90 mg/day in the absence of a compelling justification [44]. Opioid therapy should be used with caution, with periodic reassessment of the risk–benefit balance, given evidence of limited long‐term efficacy and a substantial risk of adverse effects [44]. For PHN, novel opioid formulations—e.g., transdermal oxycodone, intravenous patient‐controlled analgesia with hydromorphone, and sustained‐release tramadol (bilayer: 65% sustained‐release/35% immediate‐release)—have shown favorable safety profiles in clinical trials; however, further study is needed before widespread clinical adoption [74].

##### 3.4.3.3 Targeted Drug Delivery

The 2019 *CAMP* classifies TDD as the “treatment of last resort” (sixth‐line therapy) for NP [44]. Current evidence for TDD in PHN is limited, consisting mainly of case reports and extrapolations from other NP conditions [44]. As an invasive intervention, TDD may cause serious complications, such as infection, catheter‐ or pump‐related malfunctions, respiratory depression, and, in rare cases, death. Therefore, TDD should be used with caution and under strict monitoring [75].

##### 3.4.3.4 Deep Brain, Motor Cortex, Peripheral Nerve, and Peripheral Field Stimulation

Deep brain and motor cortex stimulation: For PHN, available evidence consists primarily of a small number of case reports and data extrapolated from other NP conditions [76, 77]. These modalities are considered on a case‐by‐case basis for severe, treatment‐refractory disease after failure of multiple therapies [77]. Peripheral nerve and peripheral field stimulation: These less invasive approaches are better suited to focal PHN [78, 79]. The evidence base in PHN consists largely of case reports and small retrospective comparative studies; high‐quality RCTs are lacking [78, 79].

#### 3.4.4 Adjunctive Treatment

Adjunctive treatments include noninvasive neuromodulation (e.g., transcutaneous electrical nerve stimulation [TENS] and high‐frequency repetitive transcranial magnetic stimulation [HF‐rTMS] over M1), psychological interventions (e.g., cognitive‐behavioral therapy and acceptance and commitment therapy), and complementary approaches (e.g., massage and acupuncture) [44, 46, 47, 48, 80, 81]. These modalities generally provide short‐term pain relief or modest, consistent benefits in chronic pain and are considered safe complements to standard therapy in selected patients [44, 46, 47, 48, 80, 81]. Guideline recommendations for TENS remain inconsistent. The 2020 Deutsche Gesellschaft für Neurologie guideline does not recommend TENS for NP owing to insufficient evidence [46]. In contrast, the 2020 SFETD and SFN guidelines weakly recommend it as first‐line treatment for peripheral NP, supported by moderate‐quality evidence [47].

#### 3.4.5 Recommendations Against Treatment

At least one international guideline advises against the following approaches for NP [46, 47, 48]: (1) anticonvulsants/antiepileptics (e.g., valproate, levetiracetam, mexiletine, topiramate, lacosamide, and phenytoin), which are restricted to acute trigeminal neuralgia attacks; (2) antidepressants/neuromodulators (e.g., milnacipran and venlafaxine); (3) selective serotonin reuptake inhibitors (citalopram, escitalopram, fluoxetine, fluvoxamine, and sertraline); (4) noradrenergic and specific serotonergic antidepressants (mirtazapine); (5) nonopioid analgesics (cyclooxygenase‐2 inhibitors, paracetamol, and metamizole); (6) other agents (*N*‐methyl‐d‐aspartate receptor‐related drugs, cannabinoids, benzodiazepines, alpha‐lipoic acid, topical amitriptyline, vitamin E, and baclofen); (7) physical/neuromodulatory interventions (HF‐rTMS targeting the posterior insula or anterior cingulate cortex, pulsed magnetic field therapy, and frequency‐modulated electromagnetic neural stimulation); and (8) neurodestructive procedures.

## 4 Conclusions

PHN remains the most common and therapeutically challenging complication of HZ, particularly in older adults and other high‐risk populations, in whom it imposes a substantial and sustained burden on pain and quality of life. Despite important advances in understanding its epidemiology, pathophysiology, risk factors, and treatment, the management of established PHN remains suboptimal, with limited pain relief and variable responses to treatment. PHN management therefore requires a stratified, individualized, and multimodal approach that integrates pharmacologic, interventional, and neuromodulatory therapies based on pain severity, pain distribution, and prior treatment response. Based on current NP guidelines and available PHN‐specific evidence, we propose a tiered management framework to inform clinical decision‐making. Nevertheless, the current evidence base is limited by substantial heterogeneity, a paucity of high‐quality PHN‐specific comparative studies, and the absence of unified disease‐specific guidelines. Further high‐quality clinical studies are needed to validate and refine this tiered management framework, thereby supporting more effective individualized treatment.

## Ethics Statement

The authors have nothing to report.

## Conflicts of Interest

The authors declare no conflicts of interest.

## Supporting information


**Table S1:** List of included publications, journal information, and evidence type classification.

**Table S2:** Guideline concordance underpinning tiered classification of PHN management strategies (Tier 1–3).

## Data Availability

Data sharing not applicable to this article as no datasets were generated or analysed during the current study.
