# Supplementary material for: Postherpetic Neuralgia: Mechanisms, Risk Factors, and Stratified Management—A Narrative Review
Source: CNS Neurosci Ther. 2026 Jun 30;32(7):e71002. doi: 10.1002/cns.71002 (PMC13317692; doi:10.1002/cns.71002)
Supplement: Supplementary file 1 — Table S1: List of included publications, journal information, and evidence type classification. [file CNS-32-e71002-s001.docx]

**Supplementary Table 1. List of Included Publications, Journal Information, and Evidence Type Classification**

| **No.** | **Journal** | **JIF Quartile**  **(Year)** | **Title** | **Article Type** |
| --- | --- | --- | --- | --- |
| 1 | N Engl  J Med | Q1(2014) | Postherpetic neuralgia | narrative review |
| 2 | Infect  Dis Ther | Q1(2024) | Incidence and Burden of Herpes Zoster in Sweden: A Regional Population-Based Register Study | observational study |
| 3 | The Journal of Infection | Q1(2018) | Incidence and costs of herpes zoster and postherpetic neuralgia in German adults aged ≥50 years: A prospective study | observational study |
| 4 | Hum Vaccines | Q2(2024) | A systematic literature review of the epidemiology and burden of herpes zoster in selected locales in Asia Pacific | systematic review |
| 5 | BMJ | Q1(2019) | Herpes zoster infection | narrative review |
| 6 | Mol Psychiatry | Q1(2024) | Association of chronic pain with suicide attempt and death by suicide: a two-sample Mendelian randomization. | observational study |
| 7 | JAMA Netw Open | Q1(2025) | Prevalence of Depression and Anxiety Among Adults With Chronic Pain A Systematic Review and Meta-Analysis. | meta- analysis |
| 8 | Dermatology | Q2(2021) | Prevalence and Risk Factors of Anxiety and Depression in Patients with Postherpetic Neuralgia: A Retrospective Study | observational study |
| 9 | Pain | Q1(2024) | Co-occurrence of chronic pain and anxiety/depression symptoms in U.S. adults: prevalence, functional impacts, and opportunities. | observational study |
| 10 | Clinicoecon Outcomes Res | Q2/Q3(2019) | Patient and economic burdens of postherpetic neuralgia in China | observational study |
| 11 | Infect Dis Ther | Q1(2022) | Meta-Regression of Herpes Zoster Incidence Worldwide | meta- analysis |
| 12 | Clin Infect Dis | Q1(2021) | Herpes Zoster and Postherpetic Neuralgia: Changing Incidence Rates From 1994 to 2018 in the United States. | observational study |
| 13 | Open Forum Infectious Diseases | Q1/Q2(2025) | The Economic Burden of Herpes Zoster in Individuals Aged 50 Years or Older and Those With Underlying Conditions in Italy. | observational study |
| 14 | The Journal of Infection | Q1(2018) | Impact of postherpetic neuralgia: A six year population-based analysis on people aged 50 years or older. | observational study |
| 15 | BMC Infect Dis | Q2(2017) | Varicella zoster virus-associated morbidity and mortality in Africa – a systematic review. | systematic review |
| 16 | Open Forum Infectious Diseases | Q1/Q2(2024) | Incidence of Herpes Zoster and Postherpetic Neuralgia and Herpes Zoster Vaccination Uptake in a US Administrative Claims Database | observational study |
| 17 | JAMA | Q1(2019) | Effect of Recombinant Zoster Vaccine on Incidence of Herpes Zoster After Autologous Stem Cell Transplantation | randomized clinical trial |
| 18 | PLoS One | Q2(2021) | Herpes zoster in older adults in Ontario, 2002-2016: Investigating incidence and exploring equity | observational study |
| 19 | Pain | Q1(2017) | Peripheral neuropathic pain: a mechanism-related organizing principle based on sensory profiles | observational study |
| 20 | Pain Ther | Q2(2019) | The Epidemiology of Herpes Zoster and Postherpetic Neuralgia in China: Results from a Cross-Sectional Study | observational study |
| 21 | BMC Public Health | Q1(2018) | Epidemiology and long-term disease burden of herpes zoster and postherpetic neuralgia in Taiwan: a population-based, propensity score-matched cohort study | observational study |
| 22 | Brain | Q1(2024) | HSV-1 reactivation results in post-herpetic neuralgia by upregulating Prmt6 and inhibiting cGAS-STING | laboratory experimental studies |
| 23 | Journal of Medical Virology | Q1(2024) | Varicella zoster virus-induced autophagy in human neuronal and hematopoietic cells exerts antiviral activity | laboratory experimental studies |
| 24 | Cell Death Dis | Q1(2024) | Varicella zoster virus glycoprotein E facilitates PINK1/Parkin-mediated mitophagy to evade STING and MAVS-mediated antiviral innate immunity | laboratory experimental studies |
| 25 | Front Mol Neurosci | Q2(2018) | A-Type K(V) Channels in Dorsal Root Ganglion Neurons: Diversity, Function, and Dysfunction | narrative review |
| 26 | Front Mol Neurosci | Q2(2023) | Ectopic expression of Nav1.7 in spinal dorsal horn neurons induced by NGF contributes to neuropathic pain in a mouse spinal cord injury model | laboratory experimental studies |
| 27 | Pain | Q1(2023) | Cutaneous nerve fiber and peripheral Nav1.7 assessment in a large cohort of patients with postherpetic neuralgia | observational study |
| 28 | Journal of Neuroinflammation | Q1(2021) | Inhibiting BDNF/TrkB.T1 receptor improves resiniferatoxin-induced postherpetic neuralgia through decreasing ASIC3 signaling in dorsal root ganglia | laboratory experimental studies |
| 29 | Front Immunol | Q1(2022) | Dynamic Immune Landscape and VZV-Specific T Cell Responses in Patients With Herpes Zoster and Postherpetic Neuralgia | observational study |
| 30 | Proc Natl Acad Sci U S A | Q1(2018) | Macrophage angiotensin II type 2 receptor triggers neuropathic pain | laboratory experimental studies |
| 31 | Inflammopharmacology | Q1/Q2(2023) | Microglia polarization in nociplastic pain: mechanisms and perspectives | narrative review |
| 32 | Biological Psychiatry | Q1(2023) | Dose-Dependent Augmentation of Neuroplasticity-Based Auditory Learning in Schizophrenia: A Double-Blind, Placebo-Controlled, Randomized, Target Engagement Clinical Trial of the NMDA Glutamate Receptor Agonist d-serine | randomized clinical trial |
| 33 | NeuroImage | Q1(2020) | Deficits in ascending and descending pain modulation pathways in patients with postherpetic neuralgia | observational study |
| 34 | Front Mol Neurosci | Q2(2023) | Genetic polymorphisms of PRKAA1 (AMPKα1) and postherpetic pain susceptibility: Multicenter, randomized control, and haplotype analysis study | observational study |
| 35 | Mol Pain | Q1/Q3(2021) | Genome-wide association study identifies candidate loci associated with chronic pain and postherpetic neuralgia | observational study |
| 36 | Biomed Pharmacother | Q1(2023) | Shouhui Tongbian Capsules induce regression of inflammation to improve intestinal barrier in mice with constipation by targeted binding to Prkaa1: With no obvious toxicity | laboratory experimental studies |
| 37 | Nature | Q1(2025) | SLC45A4 is a pain gene encoding a neuronal polyamine transporter | laboratory experimental studies |
| 38 | Int J Infect Dis | Q1(2024) | Association of the incidence of postherpetic neuralgia with early treatment intervention of herpes zoster and patient baseline characteristics: A systematic review and meta-analysis of cohort studies | meta- analysis |
| 39 | Front Mol Neurosci | Q2(2024) | Risk factors for poor prognosis in patients with zoster-associated neuralgia who underwent interventional pain management | observational study |
| 40 | Pain Physician | Q2(2020) | Delayed Initiation of Supplemental Pain Management is Associated with Postherpetic Neuralgia: A Retrospective Study | observational study |
| 41 | Front Genet | Q2(2024) | Genetic insights into the gut microbiota, herpes zoster, and postherpetic neuralgia: a bidirectional two-sample Mendelian randomization study | observational study |
| 42 | Front Neurol | Q2(2024) | Investigating the causal effect of various metabolites on postherpetic neuralgia: a Mendelian randomization study | observational study |
| 43 | Pain Ther | Q1(2024) | Identifying and Evaluating Biological Markers of Postherpetic Neuralgia: A Comprehensive Review | narrative review |
| 44 | Neurol Res Pract | Q2(2020) | Guideline "diagnosis and non interventional therapy of neuropathic pain" of the German Society of Neurology (deutsche Gesellschaft fur Neurologie) | international guideline |
| 45 | Pain Medicine | Q1(2019) | A Comprehensive Algorithm for Management of Neuropathic Pain | evidence-based review |
| 46 | Rev Neurol | Q3(2020) | Pharmacological and non-pharmacological treatments for neuropathic pain: Systematic review and French recommendations | international guideline |
| 47 | Lancet Neurol | Q1(2025) | Pharmacotherapy and non-invasive neuromodulation for neuropathic pain: a systematic review and meta-analysis | evidence-based review |
| 48 | NICE Guideline (CG173) | NR | Neuropathic pain in adults: pharmacological management in non-specialist settings. | international guideline |
| 49 | COCHRANE DB Syst Rev | Q1(2017) | Gabapentin for chronic neuropathic pain in adults. | Cochrane Database Syst Rev |
| 50 | COCHRANE DB Syst Rev | Q1(2019) | Pregabalin for neuropathic pain in adults | Cochrane Database Syst Rev |
| 51 | Pain | Q1(2019) | Mirogabalin for the management of postherpetic neuralgia: a randomized, double-blind, placebo-controlled phase 3 study in Asian patients | randomized clinical trial |
| 52 | JAMA Dermatol | Q1(2024) | Efficacy and Safety of Crisugabalin (HSK16149) in Adults with Postherpetic Neuralgia | randomized clinical trial |
| 53 | Front Neurol | Q2/Q3(2024) | Efficacy and safety of subcutaneous injection of botulinum toxin in the treatment of Chinese postherpetic neuralgia compared to analgesics: a systematic review of randomized controlled trials and meta-analysis | meta- analysis |
| 54 | Dermatol Ther | Q1(2023) | Randomized, Placebo-Controlled, Multicenter Clinical Study on the Efficacy and Safety of Lidocaine Patches in Chinese Patients with Postherpetic Neuralgia | randomized clinical trial |
| 55 | COCHRANE DB Syst Rev | Q1(2017) | Topical capsaicin (high concentration) for chronic neuropathic pain in adults | Cochrane Database Syst Rev |
| 56 | Clinical Journal of Pain | Q1/Q2(2020) | Efficacy of Pulsed Radiofrequency in Herpetic Neuralgia: A Meta-Analysis of Randomized Controlled Trials. | meta- analysis |
| 57 | Neuromodulation | Q1(2025) | From Short-Term Relief to Long-Term Management: A Meta-Analysis of Temporary Spinal Cord Stimulation and Pulsed Radiofrequency in Postherpetic Neuralgia. | meta- analysis |
| 58 | Front Neurol | Q2/Q3(2025) | Efficacy and safety of short-term spinal cord stimulation and pulsed radiofrequency in the treatment of postherpetic neuralgia: a meta-analysis | meta- analysis |
| 59 | JAMA Intern Med | Q1(2019) | A Clinical Overview of Off-label Use of Gabapentinoid Drugs | narrative review |
| 60 | Pain Ther | Q1(2022) | A Meta-analysis of Randomized Controlled Trials Comparing the Efficacy and Safety of Pregabalin and Gabapentin in the Treatment of Postherpetic Neuralgia | meta- analysis |
| 61 | Drugs | Q1(2023) | Gabapentinoids for Pain: A Review of Published Comparative Effectiveness Trials and Data Submitted to the FDA for Approval | narrative review |
| 62 | COCHRANE DB Syst Rev | Q1(2017) | Antidepressants for pain management in adults with chronic pain: a network meta-analysis. | Cochrane Database Syst Rev |
| 63 | CNS Neurosci Ther | Q1(2025) | Comparison of Duloxetine Supplemented With Pregabalin and Amitriptyline Supplemented With Pregabalin for the Treatment of Postherpetic Neuralgia: A Double-Blind, Randomized Crossover Trial | randomized clinical trial |
| 64 | Postgraduate medicine | Q1(2020) | Utility of lidocaine as a topical analgesic and improvements in patch delivery systems. | narrative review |
| 65 | Diabetes & Metabolism Journal | Q1(2023) | Pharmacological and Nonpharmacological Treatments for Painful Diabetic Peripheral Neuropathy | narrative review |
| 66 | Pain Physician | Q1(2020) | The Treatment of Topical Drugs for Postherpetic Neuralgia: A Network Meta-Analysis | meta- analysis |
| 67 | Pain | Q1(2023) | Combination pharmacotherapy for the treatment of neuropathic pain in adults: systematic review and meta-analysis | meta- analysis |
| 68 | Br J Anaesth. | Q1(2021) | Association between opioid-related deaths and prescribed opioid dose and psychotropic medicines in England: a case-crossover study | observational study |
| 69 | Pain Ther | Q1(2025) | Multimodal Therapies for the Treatment of Neuropathic Pain: The Role of Lidocaine Patches in Combination Therapy: A Narrative Review. | narrative review |
| 70 | PeerJ | Q1/Q2(2018) | Combined therapy of pulsed radiofrequency and nerve block in postherpetic neuralgia patients: a randomized clinical trial | randomized clinical trial |
| 71 | Pain Practice | Q1(2024) | 8. Herpes zoster and post herpetic neuralgia. Pain practice : the official journal of World Institute of Pain | evidence-based review |
| 72 | Reg Anesth Pain Med | Q1(2023) | Evidence-based consensus guidelines on patient selection and trial stimulation for spinal cord stimulation therapy for chronic non-cancer pain | international guideline |
| 73 | Stereot Funct Neurosurg | Q1/Q2(2025) | Long-Term Effects of Spinal Cord Stimulation on Pain in Postherpetic Neuralgia | observational study |
| 74 | Int J Mol Sci | Q1(2023) | Investigational Drugs for the Treatment of Postherpetic Neuralgia: Systematic Review of Randomized Controlled Trials | systematic review |
| 75 | Neuromodulation | Q2(2024) | The Polyanalgesic Consensus Conference (PACC)®: Intrathecal Drug Delivery Guidance on Safety and Therapy Optimization When Treating Chronic Noncancer Pain. | international guideline |
| 76 | Oper Neurosurg | Q2/Q3(2020) | Case Series: Deep Brain Stimulation for Facial Pain | observational study |
| 77 | P M | Q1(2025) | Deep brain stimulation and motor cortex stimulation for central post-stroke pain: a systematic review and meta-analysis. | meta- analysis |
| 78 | Clin Neurol Neurosur | Q2/Q3(2014) | Peripheral field stimulation for thoracic post herpetic neuropathic pain. | observational study |
| 79 | J Pain Res | Q2(2025) | Consensus Guidelines from the American Society of Pain and Neuroscience for the Use of 60-Day Peripheral Nerve Stimulation Therapy | international guideline |
| 80 | Lancet | Q1(2025) | Opportunities for chronic pain self-management: core psychological principles and neurobiological underpinnings | narrative review |
| 81 | JAMA-J Am Med Assoc | Q1(2025) | Telehealth and Online Cognitive Behavioral Therapy-Based Treatments for High-Impact Chronic Pain A Randomized Clinical Trial | randomized clinical trial |

**Note:** For the purposes of this study, evidence-based narrative reviews are classified as “guidelines,” whereas articles published in the Cochrane Database of Systematic Reviews are classified as “systematic reviews and meta-analyses.”
