# Supplementary material for: Postherpetic Neuralgia: Mechanisms, Risk Factors, and Stratified Management—A Narrative Review
Source: CNS Neurosci Ther. 2026 Jun 30;32(7):e71002. doi: 10.1002/cns.71002 (PMC13317692; doi:10.1002/cns.71002)
Supplement: Supplementary file 2 — Table S2: Guideline concordance underpinning tiered classification of PHN management strategies (Tier 1–3). [file CNS-32-e71002-s002.docx]

**Supplementary Table 2. Guideline concordance underpinning tiered classification of PHN management strategies (Tier 1–3)**

| **Intervention** | **NeuPSIG update**  **(2025)** | **DGN guideline**  **(2020)** | **SFETD/SFN guideline**  **(2020)** | **NICE guideline**  **(2020)** | **CAMP guideline**  **(2019)** |
| --- | --- | --- | --- | --- | --- |
| Gabapentin | ☑1st ☐2nd ☐3rd ☐Not rec ☐NR | ☑1st ☐2nd ☐3rd ☐Not rec ☐NR | ☑1st ☐2nd ☐3rd ☐Not rec ☐NR | ☑1st ☐2nd ☐3rd ☐Not rec ☐NR | ☑1st ☐2nd ☐3rd ☐Not rec ☐NR |
| Pregabalin | ☑1st ☐2nd ☐3rd ☐Not rec ☐NR | ☑1st ☐2nd ☐3rd ☐Not rec ☐NR | ☐1st ☑2nd ☐3rd ☐Not rec ☐NR | ☑1st ☐2nd ☐3rd ☐Not rec ☐NR | ☑1st ☐2nd ☐3rd ☐Not rec ☐NR |
| TCAs | ☑1st ☐2nd ☐3rd ☐Not rec ☐NR | ☑1st ☐2nd ☐3rd ☐Not rec ☐NR | ☑1st ☐2nd ☐3rd ☐Not rec ☐NR | ☑1st ☐2nd ☐3rd ☐Not rec ☐NR | ☑1st ☐2nd ☐3rd ☐Not rec ☐NR |
| Duloxetine | ☑1st ☐2nd ☐3rd ☐Not rec ☐NR | ☑1st ☐2nd ☐3rd ☐Not rec ☐NR | ☑1st ☐2nd ☐3rd ☐Not rec ☐NR | ☑1st ☐2nd ☐3rd ☐Not rec ☐NR | ☑1st ☐2nd ☐3rd ☐Not rec ☐NR |
| Venlafaxine | ☑1st ☐2nd ☐3rd ☐Not rec ☐NR | ☐1st ☐2nd ☐3rd ☐Not rec ☐NR | ☑1st ☐2nd ☐3rd ☑Not rec ☐NR | ☐1st ☐2nd ☐3rd ☐Not rec ☑NR | ☑1st ☐2nd ☐3rd ☐Not rec ☐NR |
| 5% Lidocaine Patches | ☐1st ☑2nd ☐3rd ☐Not rec ☐NR | ☑1st ☐2nd ☐3rd ☐Not rec ☐NR | ☑1st ☐2nd ☐3rd ☐Not rec ☐NR | ☐1st ☐2nd ☐3rd ☐Not rec ☑NR | ☑1st ☐2nd ☐3rd ☐Not rec ☐NR |
| Capsaicin 8% Patch | ☐1st ☑2nd ☐3rd ☐Not rec ☐NR | ☐1st ☑2nd ☐3rd ☐Not rec ☐NR | ☐1st ☑2nd ☐3rd ☐Not rec ☐NR | ☐1st ☐2nd ☐3rd ☐Not rec ☑NR | ☑1st ☐2nd ☐3rd ☐Not rec ☐NR |
| BTX-A | ☐1st ☐2nd ☑3rd ☐Not rec ☐NR | ☐1st ☐2nd ☑3rd ☐Not rec ☐NR | ☐1st ☑2nd ☐3rd ☐Not rec ☐NR | ☐1st ☐2nd ☐3rd ☐Not rec ☑NR | ☐1st ☐2nd ☐3rd ☐Not rec ☑NR |
| PRF | ☐1st ☐2nd ☐3rd ☐Not rec ☑NR | ☐1st ☐2nd ☐3rd ☐Not rec ☑NR | ☐1st ☐2nd ☑3rd ☐Not rec ☐NR | ☐1st ☐2nd ☐3rd ☐Not rec ☑NR | ☐1st ☐2nd ☑3rd ☐Not rec ☐NR |
| tSCS/SCS | ☐1st ☐2nd ☐3rd ☐Not rec ☑NR | ☐1st ☐2nd ☐3rd ☐Not rec ☑NR | ☐1st ☐2nd ☑3rd ☐Not rec ☐NR | ☐1st ☐2nd ☐3rd ☐Not rec ☑NR | ☐1st ☐2nd ☑3rd ☐Not rec ☐NR |
| Opioids | ☐1st ☐2nd ☑3rd ☐Not rec ☐NR | ☐1st ☐2nd ☑3rd ☐Not rec ☐NR | ☐1st ☐2nd ☑3rd ☐Not rec ☐NR | ☐1st ☐2nd ☐3rd ☐Not rec ☑NR | ☐1st ☐2nd ☑3rd ☐Not rec ☐NR |
| Nerve blocks | ☐1st ☐2nd ☐3rd ☐Not rec ☑NR | ☐1st ☐2nd ☐3rd ☐Not rec ☑NR | ☐1st ☐2nd ☐3rd ☐Not rec ☑NR | ☐1st ☐2nd ☐3rd ☐Not rec ☑NR | ☐1st ☐2nd ☑3rd ☐Not rec ☐NR |
| TDD | ☐1st ☐2nd ☐3rd ☐Not rec ☑NR | ☐1st ☐2nd ☐3rd ☐Not rec ☑NR | ☐1st ☐2nd ☐3rd ☐Not rec ☑NR | ☐1st ☐2nd ☐3rd ☐Not rec ☑NR | ☐1st ☐2nd ☑3rd ☐Not rec ☐NR |
| PNS, PFS, DBS, MCS | ☐1st ☐2nd ☐3rd ☐Not rec ☑NR | ☐1st ☐2nd ☐3rd ☐Not rec ☑NR | ☐1st ☐2nd ☐3rd ☐Not rec ☑NR | ☐1st ☐2nd ☐3rd ☐Not rec ☑NR | ☐1st ☐2nd ☐3rd ☐Not rec ☑NR |

**Note:** To improve comparability across guidelines, we harmonized recommendation statements as follows: treatments classified as third-line or beyond (≥3rd-line) were coded as 3rd; topical therapies described only as a “weak recommendation/consider” option were coded as 2nd unless explicitly listed as 1st; and interventional/device-based therapies described only as a “weak recommendation/consider” option were coded as 3rd unless explicitly listed as 2nd. Given that the SFETD/SFN guideline issued a recommendation against venlafaxine, medications with similarly conflicting guideline recommendations were not included in the tiered classification framework.

**PHN Treatment Tiers**

**First-tier treatment:** gabapentinoids, 5% lidocaine patch, TCAs, duloxetine, and 8% capsaicin patch. **Second-tier treatment:** BTX-A, PRF, and tSCS; opioids and nerve blocks may be used for short-term analgesia. **Third-tier treatment:** opioids, TDD, and neuromodulation therapies.

**Abbreviations:** NeuPSIG, Neuropathic Pain Special Interest Group of the International Association for the Study of Pain; DGN, German Society of Neurology; SFETD/SFN, French Society for the Study and Treatment of Pain/French Society of Neurology; NICE, National Institute for Health and Care Excellence; CAMP, Comprehensive Algorithm for Management of Neuropathic Pain; TCAs, tricyclic antidepressants; BTX-A, botulinum toxin type A; PRF, pulsed radiofrequency; tSCS, temporary spinal cord stimulation; SCS, spinal cord stimulation; TDD, targeted drug delivery; PNS, peripheral nerve stimulation; PFS, peripheral field stimulation; DBS, deep brain stimulation; MCS, motor cortex stimulation; NR, not reported; Not rec, not recommended; 1st/2nd/3rd, first-/second-/third-line (3rd denotes ≥3rd-line).
